# CsPrx25, a class III peroxidase in *Citrus sinensis*, confers resistance to citrus bacterial canker through the maintenance of ROS homeostasis and cell wall lignification

**DOI:** 10.1038/s41438-020-00415-9

**Published:** 2020-12-01

**Authors:** Qiang Li, Xiujuan Qin, Jingjing Qi, Wanfu Dou, Christophe Dunand, Shanchun Chen, Yongrui He

**Affiliations:** 1 Citrus Research Institute, Southwest University/Chinese Academy of Agricultural Sciences, Chongqing, 400712 China; 2 Laboratoire de Recherche en Sciences Végétales, Université de Toulouse, CNRS, UPS, Auzeville-Tolosane, 31320 France; 3 Key Laboratory of Plant Hormones and Development Regulation of Chongqing, School of Life Sciences, Chongqing University, 401331 Chongqing, China

**Keywords:** Metabolism, Biotic

## Abstract

Citrus bacterial canker (CBC) results from *Xanthomonas citri* subsp. *citri* (*Xcc*) infection and poses a grave threat to citrus production. Class III peroxidases (CIII Prxs) are key proteins to the environmental adaptation of citrus plants to a range of exogenous pathogens, but the role of CIII Prxs during plant resistance to CBC is poorly defined. Herein, we explored the role of CsPrx25 and its contribution to plant defenses in molecular detail. Based on the expression analysis, CsPrx25 was identified as an apoplast-localized protein that is differentially regulated by *Xcc* infection, salicylic acid, and methyl jasmone acid in the CBC-susceptible variety Wanjincheng (*C. sinensis*) and the CBC-resistant variety Calamondin (*C. madurensis*). Transgenic Wanjincheng plants overexpressing *CsPrx25* were generated, and these transgenic plants exhibited significantly increased CBC resistance compared with the WT plants. In addition, the *CsPrx25*-overexpressing plants displayed altered reactive oxygen species (ROS) homeostasis accompanied by enhanced H_2_O_2_ levels, which led to stronger hypersensitivity responses during *Xcc* infection. Moreover, the overexpression of *CsPrx25* enhanced lignification as an apoplastic barrier for *Xcc* infection. Taken together, the results highlight how CsPrx25-mediated ROS homeostasis reconstruction and cell wall lignification can enhance the resistance of sweet orange to CBC.

## Introduction

Plants possess an intricate repertoire of cell-based defense systems to maintain their resistance to potentially harmful pathogens^1,2^. As an immediate pathogen recognition response, oxidative bursts produced in apoplasts induce reactive oxygen species (ROS), including superoxide (O_2_^.−^) and H_2_O_2_, as a first line of defense^3^. The current models of plant responses include ROS and other radicals as catalysts of covalent cell-wall modifications^4^, as signals for cell-death reactions^5,6^ and as regulators of resistance-associated genes^7,8^. In plants, high concentrations of ROS act to strengthen the cell wall and inhibit pathogen growth, which results in the enhancement of host resistance to pathogens via hypersensitive responses (HRs) and the modulation of gene expression via signaling molecules^9,10^. However, high accumulation of ROS can be toxic to plant cells by inhibiting plant growth and development^2^. Thus, ROS homeostasis needs to be maintained by antioxidant compounds and enzymes^11^. In plant cells, ROS are produced by NADPH oxidase resident at the cell surface, class III peroxidases (CIII Prxs, or POD) and their associated pathways, including photosynthesis, photorespiration, and respiration^12,13^. In addition, the ROS scavengers superoxide dismutase (SOD), catalase (CAT), and glutathione s-transferase (GST) cooperate with ROS producers to maintain ROS homeostasis^14^. Moreover, antioxidant enzyme activities and ROS homeostasis are regulated by important plant hormones, including jasmonic acid (JA) and salicylic acid (SA)^15–17^.

CIII Prxs are heme-binding proteins that are ubiquitously expressed in all plants and comprise large multigene families^18–21^. For example, a total of 73 CIII Prxs are present in *Arabidopsis thaliana*^22,23^, and 138, 374, 93, 94 and 72 have been found in *Oryza sativa*^24^, *Triticum aestivum*^25^, *Populus trichocarpa*^26^, *Pyrus bretschneideri*^27^ and *Citrus sinensis*^28^, respectively. CIII Prxs regulate the loosening of cell walls, lignification and suberization^29–32^ and participate in ROS and RNS metabolism during abiotic and biotic stress responses^33–35^. CIII Prxs are key to the innate resistance of many plants to both fungal and bacterial pathogens and mediate both passive and active defense mechanisms^6,36,37^, and the efficiency of this mediation determines their susceptibility to pathogenic infections^38^. Rapid ROS production is one such exemplar defense strategy that leads to O_2_^.−^ generation and H_2_O_2_ production in apoplasts. H_2_O_2_ is tightly regulated by CIII Prxs as both producers and scavengers depending on whether the enzyme participates in peroxidative cycles and hydroxylic cycles, respectively^12,13^. In French bean and tobacco plants, apoplastic CIII Prxs produce ROS and act as catalysts for covalent cell-wall modifications^4^ and cell death regulators^6^. Based on these functions of CIII Prxs, an increasing number of studies have identified links between this enzyme and pathogen attack and have improved host resistance due to CIII Prxs. Radwan and colleagues reported that bean yellow mosaic virus infection leads to increased levels of monodihydroascorbate (MDA) and H_2_O_2_ in *Vicia faba* leaves^39^. Enhanced CIII Prx and SOD activities have also been observed in leaves infected by yellow mosaic virus, which suggests that enzymatic antioxidants regulate ROS generation in response to pathogen infection^39^. Increasing the expression of a peroxidase in plants can effectively increase the resistance of the plants to disease. For example, the overexpression of *HvPrx40*^40^ and *TaPrx10*^39,41^ leads to higher levels of resistance to *Blumeria graminis* (wheat powdery mildew) in wheat (*T. aestivum*).

*Xanthomonas citri* subsp. *citri* (*Xcc*) pathogen is the causative agent of citrus bacterial canker (CBC), a known cause of citrus yield losses in an array of citrus-producing regions^42,43^. In our previous studies of the citrus transcriptomes induced by *Xcc*, we found that CIII Prxs were differentially expressed and explored the relationship between CBC and CIII Prxs, and our results revealed CsPrx25 as a potential gene for improving CBC resistance^28^. Here, we performed both a structural and functional characterization of CsPrx25. We also developed transgenic sweet orange overexpressing *CsPrx25* that displayed enhanced tolerance to CBC due to ROS homeostasis accompanied by high levels of H_2_O_2_ and high lignification of the apoplastic barrier. We herein describe the utility of transgenic plants overexpressing *CsPrx25* for enhancing CBC resistance.

## Results

### CsPrx25 encodes a CIII Prx in citrus

**Fig. 1:**
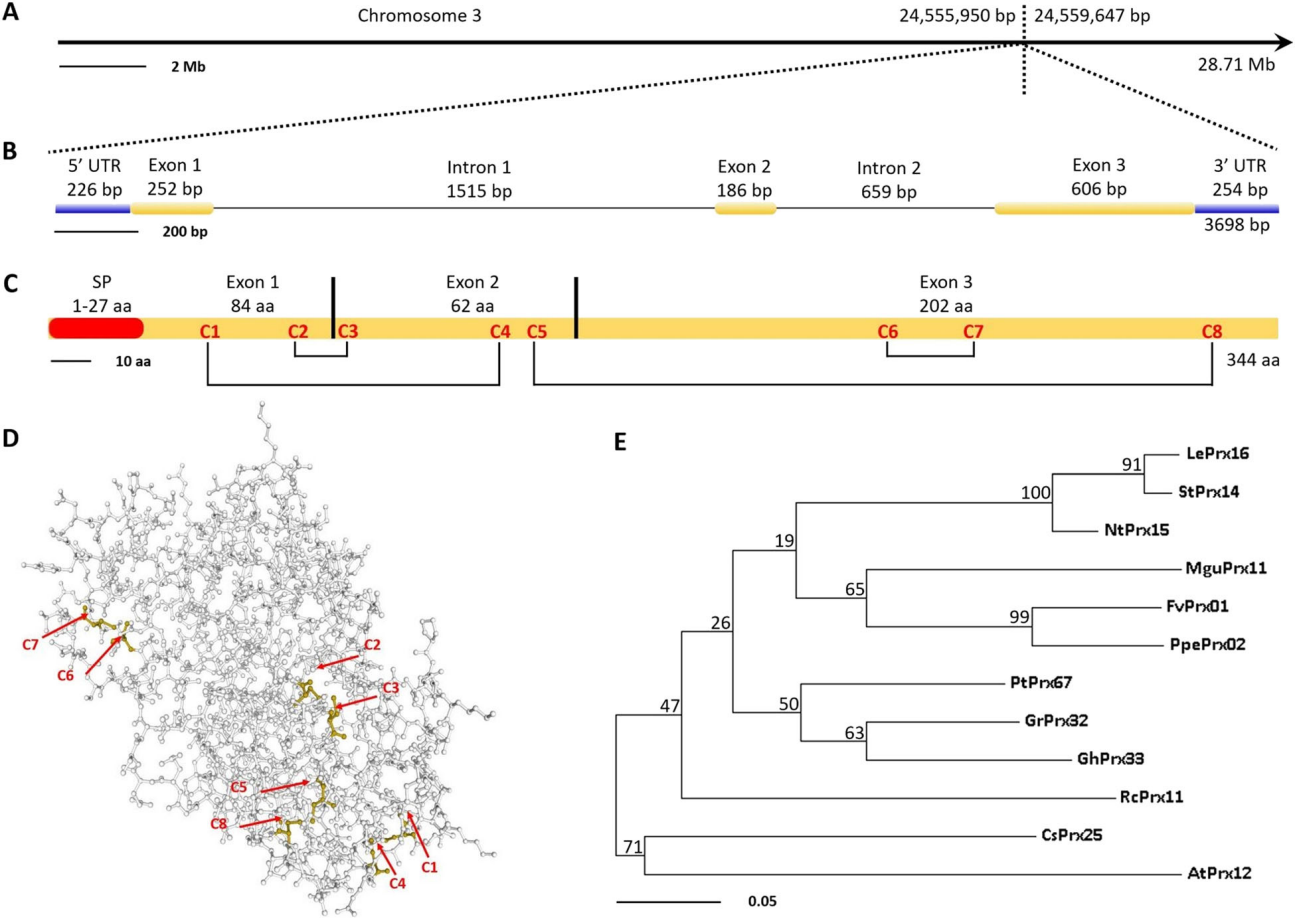
Bioinformatics features of CsPrx25. **a** *CsPrx25* chromosomal locus obtained via CAP. **b** Exons and introns of *CsPrx25* obtained through GSDS V2.0. The blue rectangles represent untranslated regions (UTRs) at the 5′ and 3′ ends; the yellow rectangles represent exons; and the blank lines represent introns. **c** Schematic of the signal peptide and cysteines (C1–C8) of CsPrx25. The disulfide bonds formed by the cysteine residues are joined by lines. The signal peptide was predicted using SignalP V4.0. **d** 3D model of CsPrx25 predicted by Phyre V2.0. The cysteine residues are labeled with red arrows and C1–C8. **e** Maximum-likelihood (ML) phylogeny of CsPrx25 and its orthologs in several organisms. The ML tree was constructed using the CIII Prx sequences of *Lycopersicon esculentum* (LePrx16), *Solanum tuberosum* (StPrx14), *Nicotiana tabacum* (NtPrx15), *Mimulus guttatus* (MguPrx11), *Fragaria vesca* (FvPrx01), *Prunus persica* (PpePrx02), *Populus trichocarpa* (PtPrx67), *Gossypium raimondii* (GrPrx32), *Gossypium hirsutum* (GhPrx33), *Ricinus communis* (RcPrx11), *A. thaliana* (AtPrx12) and *C. sinensis* (CsPrx25) with MEGA V7.0 (bootstrap: 500, Poisson model). The clustering of the taxa is shown through the percentages of trees displaying clusters. The branches are drawn to scale, and each length is representative of the number of substitutions at each site

We amplified and sequenced the complete transcript of *CsPrx25* using cDNA from Wanjincheng leaves as the PCR template. The primary sequences were searched in PeroxiScan, which is built in RedoxiBase^44,45^. The findings revealed that *CsPrx25* belonged to the CIII Prx family (PeroxiScan accession: PS52045), a subgroup of nonanimal peroxidases (PeroxiScan accession: PS50873). The *CsPrx25* sequence was further analysed by the Blast tool built in RedoxiBase and CAP^46^, and the results revealed that *CsPrx25* was clustered with the CIII Prxs sequence ID 8898 in RedoxiBase and Cs3g21730 in CAP due to 100 and 98% sequence similarities, respectively. CsPrx25 is a 344-residue CIII Prx (molecular weight: 38.06 kD; isoelectric point: 8.55) present on chromosome 3 of *C. sinensis* (Fig. 1a) that possesses two introns (1515 bp and 659 bp, respectively) (Fig. 1b). The N-terminus of CsPrx25 contains a signal peptide of 27 residues that is required for correct trafficking to the apoplast. Throughout the sequence, eight cysteine residues were detected (C1–C8) (Fig. 1c), and these form a total of four disulfide bonds (DB) that maintain thermal stability. These 4-DB structures are common to almost all plant CIII Prxs and impart distinction from ascorbate and other plant peroxidases^47^. The three-dimensional (3D) structures also showed that the cysteines that form disulfide bonds are close to each other (Fig. 1d). To study the evolutive scenario of CIII Prxs between organisms, the phylogeny of CIII Prxs orthologs was assessed, and close relationships between CsPrx25 and AtPrx12 were found (Fig. 1e).

### CsPrx25 is an apoplast-localized protein that is induced by *Xcc* and phytohormones

**Fig. 2:**
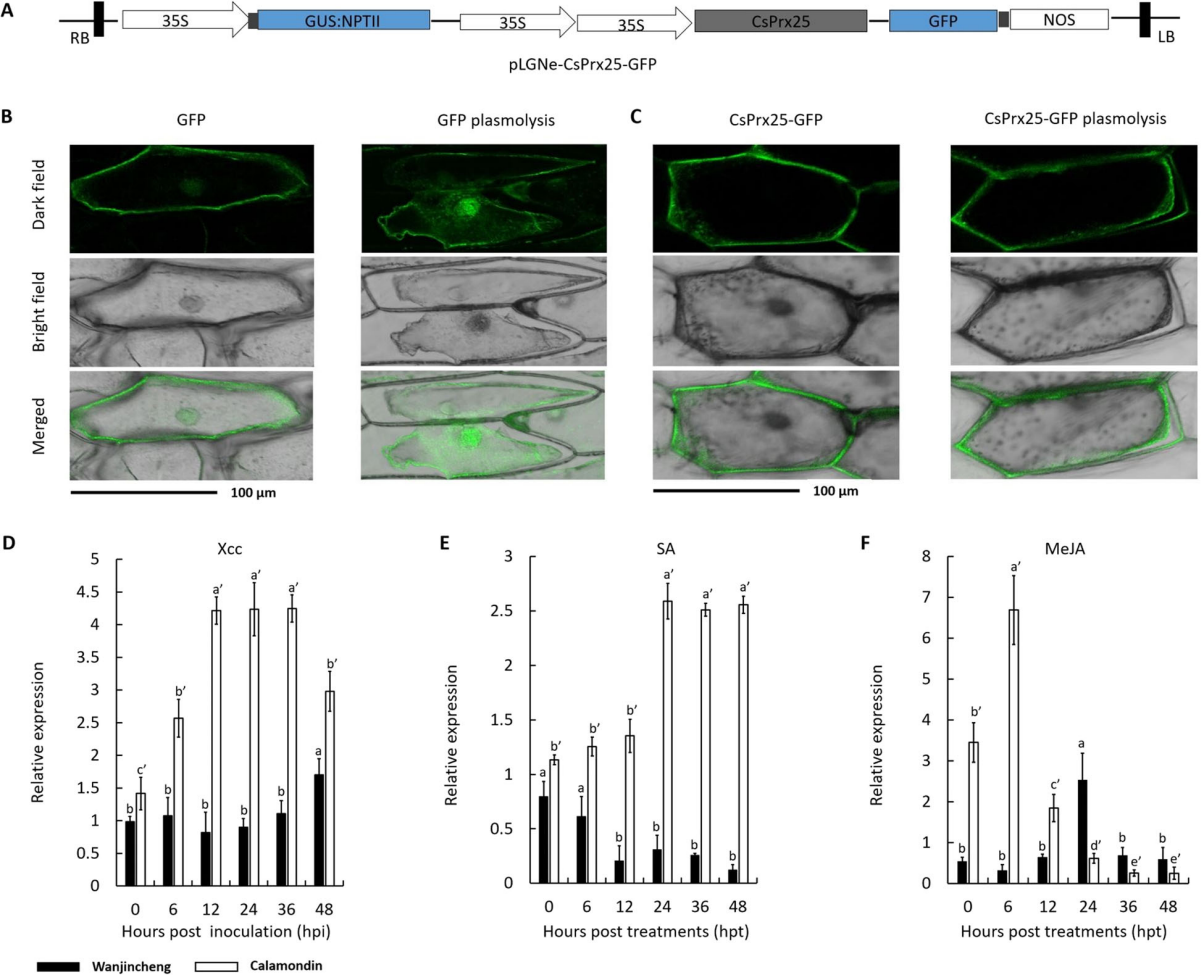
Expression profiles of *CsPrx25*. **a** Structure of the pLGNe-*CsPrx25-GFP* plasmid. LB: left border; RB: right border. The *CsPrx25* expression plasmids included the 35 S cauliflower mosaic virus promoter (35 S), the terminator of nos (NOS), and NPTII. **b** Transient expression of GFP in onion epidermal cells. **c** Transient expression of *CsPrx25-GFP*. In **b**, **c**, scale bar: 100 μm. **d** *CsPrx25* expression in response to *Xcc* in Wanjincheng (filled bars) and Calamondin (open bars). *CsPrx25* induction in response to SA (**e**) and MeJA (**f**) in Wanjincheng (filled bars) and Calamondin (open bars). The relative expression levels were measured by qRT-PCR and normalized to that of *CsActin*. In **d**–**f**, the data were analyzed using Tukey’s HSD test (*P* = 0.05; *n* = 3)

To elucidate the localization of CsPrx25, software predictions and transient expression systems were investigated. CELLO V2.5 displayed extracellular loci values of 2.46, which were larger than other loci (Supplementary Table S1). The signal peptide detected by SignalP V4.0 suggests that CsPrx25, as most of the CIII Prxs, is extracellular. To validate these predictions, the transient expression of *CsPrx25* was assessed with *35S::CsPrx25-GFP* (Fig. 2a). Relative to the controls, both cytoplasmic and nuclear fluorescence were observed before and after plasmolysis (Fig. 2b). In epidermal onion cells, CsPrx25-GFP showed robust cell surface expression (Fig. 2c), confirming that CsPrx25 localizes to apoplasts.

Pathogens and phytohormones can mediate gene expression changes that occur in response to plant disease^48,49^. In Calamondin, *CsPrx25* was upregulated, and maximal expression (~5-fold) was observed at 36 hpi. In contrast, Wanjincheng *CsPrx25* showed little-to-no expressional changes in response to *Xcc* infection (Fig. 2d). To detect the effect of drought during in vitro inoculation, we tested the inducibility of *CsPrx25* under drought stress. The results indicated that *CsPrx25* was hardly induced by drought in both varieties, which indicated that it was specifically induced by *Xcc* (Supplementary Fig. S1). *CsPrx25* is therefore likely to represent an *Xcc* resistance gene. To reveal the molecular mechanisms through which *CsPrx25* mediates disease resistance, *CsPrx25* transcripts were assessed in SA- and MeJA-treated leaves. The expression of *CsPrx25* rapidly increased in Calamondin in response to SA. In contrast, *CsPrx25* expression was downregulated in Wanjincheng (Fig. 2e). The expression of *CsPrx25* induced by MeJA increased and then decreased over time in both Wanjincheng and Calamondin, and the times to maximal expression in these varieties was different (Wangjincheng: 24 hpt vs Calamondin: 6 hpt) (Fig. 2f). The different expression patterns of *CsPrx25* induced by phytohormones indicate the different roles of *CsPrx25* in disease resistance signaling in Calamondin and Wanjincheng.

### *CsPrx25* overexpression in sweet orange induces resistance to CBC

**Fig. 3:**
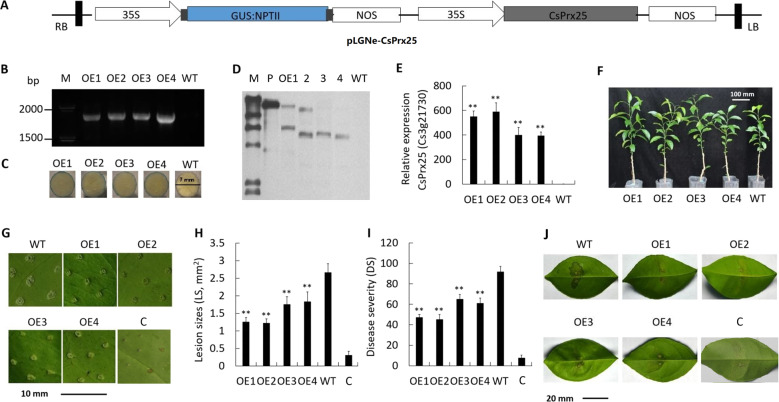
The overexpression of *CsPrx25* in sweet orange confers CBC resistance. **a** Structure of the pLGNe-*CsPrx25* plasmid used for the overexpression assays. **b** Validation of the transgenic plants by PCR. **c** Confirmation of the transgenic lines by the GUS assay (diameter of leaf discs: 7 mm). **d** Southern blot of transgenic and WT lines. P: pLGNe-*CsPrx25* plasmid. Exposure period: 1 h. **e** *CsPrx25* expression assessed by qRT-PCR with CsActin as an internal control. **f** Transgenic lines and their phenotypes. Scale bar: 100 mm. **g** The symptoms of OE and WT plants inoculated with *Xcc* were assessed and imaged at 10 dpi. Scale bar: 10 mm. Lesion size (LS) **h** and disease severity (DS) **i** of the OE and WT plants at 10 dpi. In **e**, **h, i**, the data were normalized to the data from the WT plants and compared using Tukey’s HSD test (*P* = 0.05, *n* = 3). **j** Infiltration assays for measuring CBC resistance. *Xcc* was found to induce disease-related symptoms after 10 dpi. Scale bar: 20 mm. In **g–j**, OE1–4 represent the four transgenic Wangjincheng plants; WT and C represent the wild-type Wanjincheng and Calamondin plants, respectively

Transgenic citrus constructs overexpressing *CsPrx25* were used to fully dissect the role of CsPrx25 during *Xcc* resistance. *CsPrx25* was overexpressed using exogenous expression plasmids driven by the 35S promoter (Fig. 3a). The generation of four *CsPrx25*-overexpressing plants 1–4 (OE1–OE4) that successfully integrated *CsPrx25* was confirmed by qRT-PCR, GUS assay and Southern blot. Through PCR, we detected an 1874-bp fragment that was not present in the wild-type (WT) lines (Fig. 3b), and the GUS assay revealed blue color on the periphery of the leaf discs (Fig. 3c). As determined by Southern blot, OE1 and OE2 contain two copies of *CsPrx25*, and OE3 and OE4 harbor only one copy (Fig. 3d). We confirmed that all lines expressed high levels of *CsPrx25* (550-fold, 589-fold, 401-fold and 395-fold of the WT levels, respectively) by qRT-PCR analysis (Fig. 3e). According to the Southern blot assay, a certain positive correlation exists between copy number and expression (Fig. 3d). With respect to phenotypes, the four transgenic lines showed normal growth rates compared with the WT lines (Fig. 3f). Acupuncture is an effective method for quantitatively assessing the resistance to CBC and can be used to accurately quantify CBC resistance, which would allow the assessment and comparison of resistance between varieties^50,51^. To assess the CBC resistance of *CsPrx25*-OE plants, in vitro assays were performed with acupuncture inoculation at 10 dpi. Smaller lesion sizes, which are indicative of less-severe symptoms, were observed in the OE leaves compared with the WT leaves (Fig. 3g). This finding suggested that *Xcc* pustules are reduced by *CsPrx25* overexpression, and OE2 showed the highest levels of resistance. Compared with the WT plants, OE2 showed smaller lesions (45.8% of the WT levels), OE1 exhibited comparable lesions (47.0% of the WT levels), and OE3 and OE4 displayed larger lesions (65.8% and 68.8% of the WT levels) (Fig. 3h). The disease severity decreased by 29.2% (OE3) to 50.7% (OE2) in the OE plants compared with WT plants (Fig. 3i). Using infiltration assays after 10 dpi, symptoms of canker (including pustules) were observed in then WT lines, but these symptoms were markedly reduced in the OE plants (Fig. 3j). We therefore conclude that *CsPrx25* overexpression enhances *Xcc* resistance in the OE transgenic citrus lines.

### *CsPrx25* overexpression modulates the enzymatic antioxidant system

**Fig. 4:**
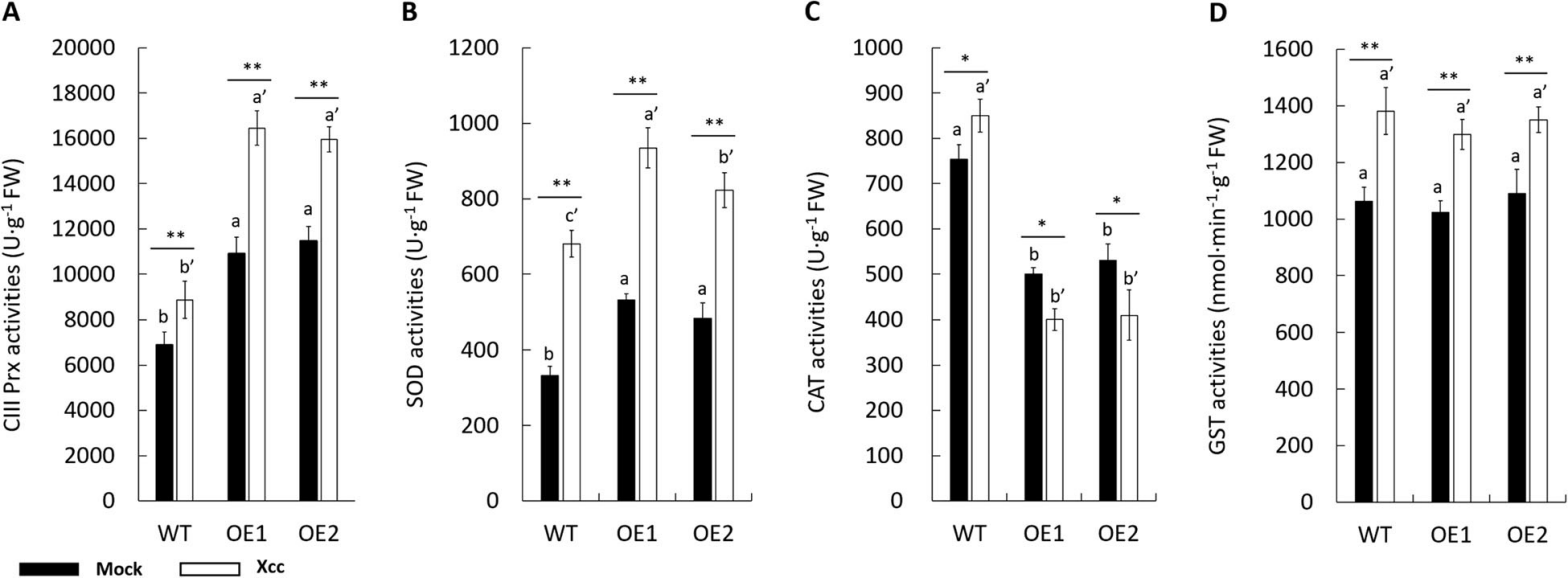
*CsPrx25* overexpression modulates the enzymatic antioxidant system. CIII Prx (**a**), SOD (**b**), CAT (**c**) and GST (**d**) in both OE and WT plants inoculated with mock (ddH_2_O) (filled bars) and *Xcc* (open bars) for 12 h. FW: fresh weight. In **a**–**d**, the values were compared to those found for the WT lines. The differences between the mock- and *Xcc*-infected samples were analyzed using Fisher’s LSD test, *P < 0.05; **P < 0.01. Tukey’s HSD test was used to compare the WT and OE plants (*P* = 0.05; *n* = 3)

Plants possess a well-developed ROS homeostasis enzymatic system that efficiently regulates the ROS levels, and this system includes CIII Prx, SOD, CAT and GST^38,52^. To assess the changes in the antioxidant system following the induction of CsPrx25-mediated resistance to *Xcc*, the antioxidant activity in transgenic lines in these lines was compared with that in the WT plants at 12 hpi. OE plants with higher resistance (OE1 and OE2) to CBC were selected for analysis. The activities of both CIII Prx and SOD were upregulated by *CsPrx25* overexpression (Fig. 4a, b). The overexpression of *CsPrx25* conferred antioxidant defenses and led to the induction by *Xcc* infection. In contrast to CIII Prx and SOD, the activities of CAT in OE plants were downregulated, and the *Xcc*-induced profiles were altered compared with those observed in the WT plants (Fig. 4c). In contrast to SOD, CIII Prx and CAT, the overexpression of *CsPrx25* did not affect the activity of GST compared with that found in the WT plants (Fig. 4d).

### *CsPrx25* overexpression establishes ROS homeostasis to confer a more sensitive HR to *Xcc* infection

**Fig. 5:**
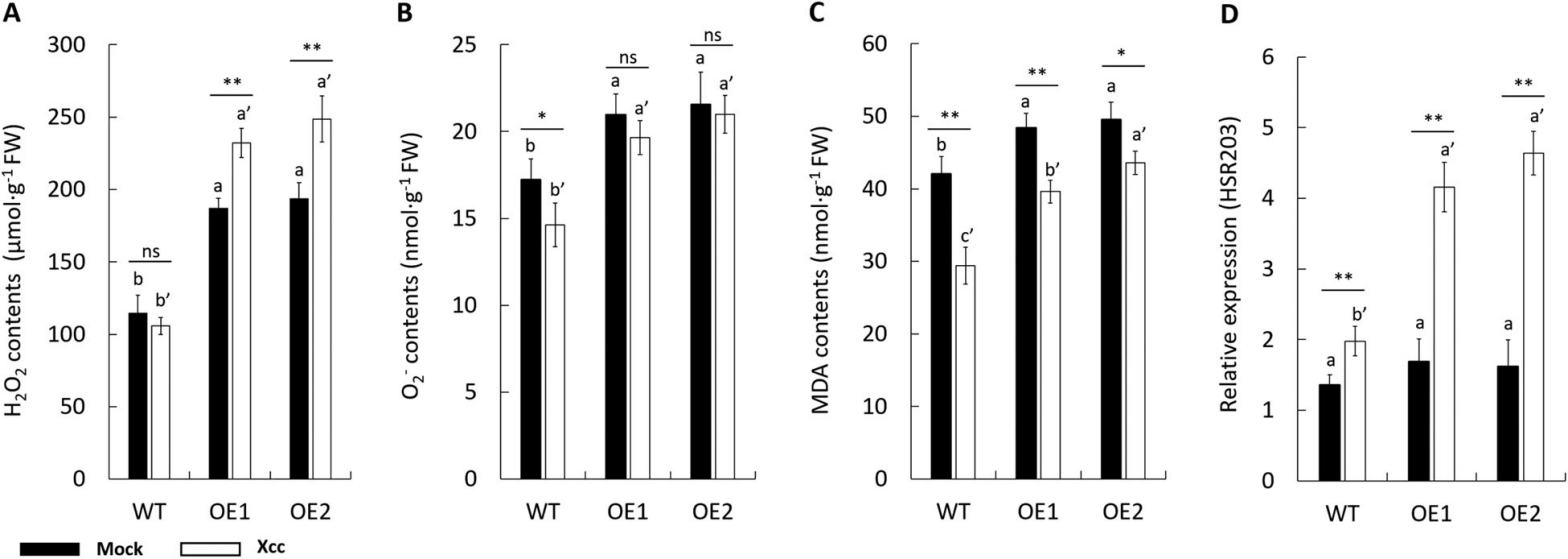
CsPrx25 reconstructs ROS homeostasis and imparts HR sensitivity during *Xcc* infection. The levels of H_2_O_2_ (**a**), O_2_^.−^ (**b**) and MDA (**c**) in OE and WT plants were assayed at 12 h after mock inoculation (ddH_2_O; filled bars) and *Xcc* infection (open bars). In **a**–**c**, FW: fresh weight. **d** *CsHSR203* transcript levels in WT vs. OE plants at 12 h after mock (ddH_2_O) (filled bars) or *Xcc* inoculation (open bars). The data were normalized to the *CsActin* levels. In **a**–**d**, the differences between the mock- and *Xcc*-infected samples were analyzed by Fisher’s LSD test, *P < 0.05; **P < 0.01. The differences between the OE and WT plants were analyzed using Tukey’s HSD test (*P* = 0.05; *n* = 3)

In response to pathogen infection, ROS production intricately controls many responses, including apoptotic cell death and oxidative damage^53,54^. To confirm the involvement of ROS homeostasis in CsPrx25-mediated *Xcc* resistance, the levels of H_2_O_2_ and O_2_^.−^ in WT vs. *CsPrx25*-OE lines were assessed. We observed higher levels of H_2_O_2_ in the OE lines. Of interest, *Xcc* infection did not significant change the levels of H_2_O_2_ in WT plants but increased these levels in the OE lines (Fig. 5a). This finding suggested that *CsPrx25* overexpression not only increased the levels of H_2_O_2_ but also reversed the inducible patterns of H_2_O_2_ during *Xcc* infection. The levels of O_2_^.−^ also increased in response to *CsPrx25* overexpression (Fig. 5b). The cell membrane is first affected by lipid peroxidation, and MDA is the final product^55^. A spectroscopic analysis of the transgenic and WT plants revealed elevated levels of MDA, and these levels were modestly reduced in response to *Xcc* infection (Fig. 5c), which indicate lower levels of damage following *Xcc* infection in both the transgenic and WT plants. These data indicate that Wanjincheng has the ability to suppress the oxidative damage caused by *Xcc* infection, and this suppression is strengthened by *CsPrx25* overexpression. H_2_O_2_ is a key mediator of an early HR. Because *CsPrx25* overexpression regulates H_2_O_2_ modulation, the immediate question was whether the HR is also altered in the transgenic plants. To investigate the relationship between the increased CBC resistance induced by CsPrx25 and HR, we assessed the HR of the transgenic plants before and after *Xcc* infection. The expression of the HR marker gene, *CsHSR203*^56–58^ was significantly upregulated in the transgenic plants infected with *Xcc* but only modestly increased in the *Xcc*-infected WT plants. No obvious changes in the expression of *CsHSR203* were observed between the transgenic and WT plants in the absence of *Xcc* infection (Fig. 5d). We therefore conclude that the transgenic plants are more sensitive to a HR following *Xcc* infection, which increases the early resistance of transgenic plants to CBC.

### *CsPrx25* overexpression enhances lignification as an apoplastic barrier for *Xcc* infection

**Fig. 6:**
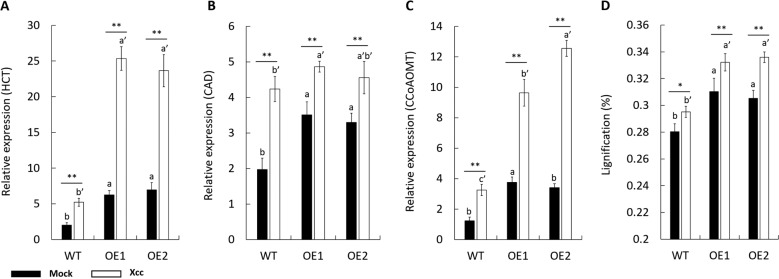
*CsPrx25* positively regulates cell wall lignin biosynthesis. Transcripts of the genes involved int he phenylpropanoid pathway, including *HCT* (**a**), *CAD6* (**b**) and *CCoAOMT* (**c**), in WT vs. OE plants at 12 h after mock (ddH_2_O) inoculation (filled bars) or *Xcc* (open bars) infection. **d** Spectroscopic analysis of lignin at 12 hpi. Leaves of OE1, OE2 and WT plants were sampled for lignification analysis. In **a**–**d**, the values were statistically compared with those found for the WT lines. The differences between the mock- and *Xcc*-infected samples were analyzed by Fisher’s LSD test, *P < 0.05; **P < 0.01. The differences between the OE and WT plants were analyzed using Tukey’s HSD test (*P* = 0.05; *n* = 3)

CIII Prxs regulate cell wall lignification, which suggests a direct role of CIII Prxs on the cell walls of plants^6,59–61^. To investigate the effects of lignification on *Xcc* resistance in transgenic plants, the role of CsPrx25 in lignification was assessed. The transcript levels of lignin biosynthetic genes, namely, hydroxycinnamoyl transferase (*CsHCT*, CAP ID: Cs1g14450), cinnamyl alcohol dehydrogenase (*CsCAD*, CAP ID: Cs1g20590) and caffeoyl-CoA O-methyltransferase (*CsCCoAOMT*, CAP ID: Cs4g13430), were elevated in the leaves of the transgenic lines after mock inoculation and *Xcc* infection (Fig. 6a–c). These findings highlight the role of CsPrx25 in lignin biosynthesis. All the data were confirmed through lignin assays, which showed higher values in the transgenic compared with the WT plants (Fig. 6d). These data therefore reflect the role of CsPrx25 in the polymerization of lignin during its biosynthesis and highlight its importance in CBC resistance through enhanced lignification.

### CsPrx25 enhances CBC resistance, and this effect is associated with ROS homeostasis reconstruction and lignification

**Fig. 7:**
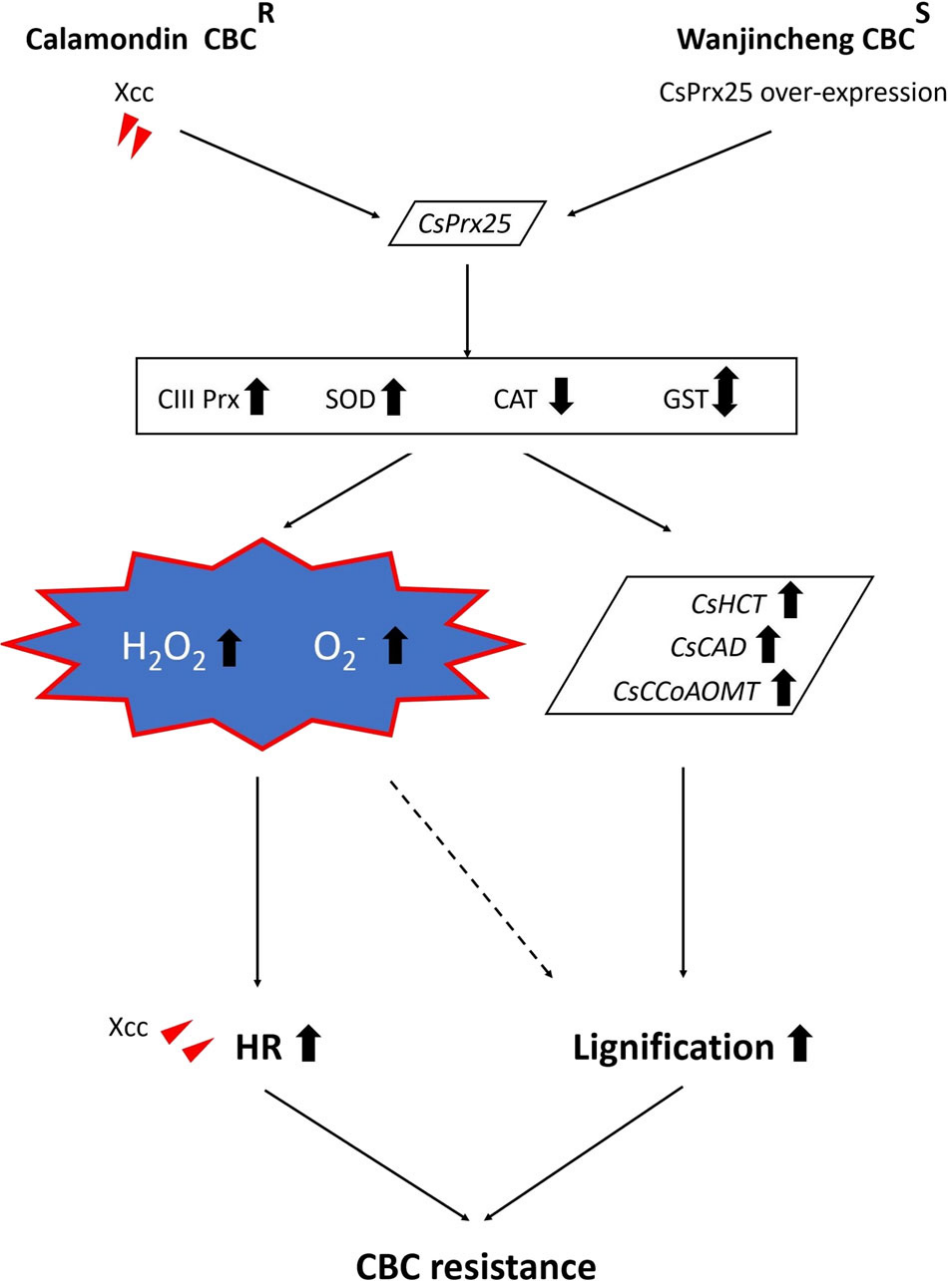
Hypothetical model of the molecular mechanisms mediating improved CBC resistance through CsPrx25. Increases, decreases and no changes are represented by up arrows, down arrows, and double arrows, respectively. The genes involved in this model are written in the trapezoids and in italics. Antioxidants are shown in rectangles. The ROS content is written in starburst

*CsPrx25* overexpression confers ROS homeostasis to the transgenic lines through modulation of the enzymatic antioxidant system (Figs. 4–5). The levels of lignin were also higher in the transgenic lines than in the WT plants, and some lignin biosynthetic genes were more highly expressed in the transgenic lines (Fig. 6). Based on these results, we proposed a model to explain how Calamondin and *CsPrx25*-OE transgenic Wanjincheng acquired CBC resistance (Fig. 7). In Calamondin, *Xcc* infection improves the levels of *CsPrx25*, and this effect enhances the H_2_O_2_ levels and HR sensitivity and induces lignification, resulting in CBC resistance. The overexpression of *CsPrx25* in CBC-susceptible Wanjincheng establishes ROS homeostasis, and higher H_2_O_2_ levels confer HR sensitivity in response to *Xcc* infection. In the transgenic plants, *CsPrx25* overexpression also enhanced lignin biosynthesis, reinforcing the apoplastic barrier for *Xcc* infection. Through these two mechanisms, CsPrx25 promotes CBC resistance.

## Materials and methods

### Plants, bacteria and growth conditions

All the plants were obtained from the National Citrus Germplasm Repository. Wanjincheng (*C. sinensis*) was used for gene transformations. All the plants were grown at 28 °C in a greenhouse. The *Xcc* variants were derived from citrus leaves that are susceptible to natural infections. The *Xcc* cultures were grown at 28 °C in peptone-yeast extract-malt extract containing 1.5% (w/v) d-glucose.

### In silico characterization of CsPrx25

The complete transcript sequence of *CsPrx25* was amplified from Wanjincheng leaves using the primers F_clone_ (ATGGCAACTGCTTCAGCTTCT) and R_clone_ (TTAGATAATCCCAGACCAAGC). PeroxiScan was used for the family classification of CsPrx25^20^. Blast tools built in RedoxiBase^20,44^, CAP^46^, CitGVD^62^ and SMART^63^ were used to reconfirm the sequence of *CsPrx25* retrieved by PCR. The chromosomal loci and the locations of exons and introns were defined using GSDS V2.0^64^ based on the genome assembly of *C. sinensis* in CAP. SignalP V4.0^65^ was used for signal peptide predictions, and CELLO V2.5^66^ was used for cellular localization prediction. Phyre V2.0^67^ was used for the 3D assessments of CsPrx25. The gene, protein and coding sequences (CDSs) of *CsPrx25* are shown in Table S2.

### Transient expression of GFP-tagged *CsPrx25*

The coding sequence (CDS) of CsPrx25 lacking a stop codon was amplified with flanking restriction sites using the primers F_SC_ (CGGGGTACCATGGCTGTTCATCAACATTATCTGG) (*Kpn*I) and R_SC_ (TCCCCCGGGTCACTGGTTTGAAATTAAAGGATCT) (*Sma*I), digested, recovered and cloned into pLGNe-*GFP* driven by the 35S promoter to construct the recombinant plasmid pLGNe-*CsPrx25-GFP*. The pLGNe-*CsPrx25-GFP* plasmid encodes a fusion protein composed of CsPrx25 and GFP. The plasmids were heat-shocked into *Agrobacterium* EHA105. The transformed EHA105 was infiltrated into onion epidermal cells, and the GFP fluorescence signals were observed at 48 hpi by laser-scanning confocal microscopy (LSM 510 Meta, Zeiss).

### Treatments with *Xcc* and phytohormones

The expression of *CsPrx25* in excised leaves maintained in culture plates for 16 h of light and 8 h of darkness was assessed. Diluted *Xcc* (OD600: 0.8) was inoculated onto the leaves at 28 °C, and after defined durations, the expression of *CsPrx25* was assessed by qRT-PCR. For phytohormone assessments, leaf discs were soaked in 10 μmol L^-1^ SA or 100 μmol L^-1^ MeJA and collected for qRT-PCR assays of exogenous phytohormones. The primers used for *CsPrx25* detection were F_RT_ (CCCCACTTCGGATTCCAACA) and R_RT_ (CAACCCCTGTCGGTTCATCA).

### Overexpression vector construction and plant transformation

For the generation of overexpression lines, full-length *CsPrx25* was PCR amplified using F_OEC_ (GGGGTACCATGGCAACTGCTTCAGCTTC) and R_OEC_ (CGGGATCCTTAGATAATCCCAGACCAAGCC) and cloned into pLGNe to yield the recombinant plasmid pLGNe-*CsPrx25*. Wanjincheng shoot transformations were performed using *Agrobacterium tumefaciens* as previously described by Li and He^48,50^.

### Validation of the transgenic lines by PCR and GUS assays

PCR assays were used to confirm the presence of the transgenic gene with the primers F_OED_ (CGACACGCTTGTCTACTCCA) and R_OED_ (CGGGATCCTTAGATAATCCCAGACCAAGCC). GUS activity was assessed through histochemical analysis^48,51^.

### Southern blot assay

Total genomic DNA (gDNA) was extracted from the leaves of the transgenic plants and WT plants using a CTAB kit (Zoonbio, China). The gDNA was fragmented using the restriction enzyme *EcoR*I, and the DNA fragments were separated on a 0.7% agarose gel and transferred to a Hybond-N^+^ membrane (Amersham, UK). The NPTII coding gene labeled by digoxin (DIG) was used to hybridize the membrane-bound DNA (Roche, Switzerland). The nylon membrane was then exposed using nonradioactive probe detection. In the Southern blot assay, the pLGNe-*CsPrx25* plasmid was used as the positive control.

### Assessment of CBC resistance

CBC resistance analyses were performed as previously described^48,68,69^. Briefly, six punctures were made in six healthy mature leaves of each transgenic line via 0.5-mm pins, and 1 µL of each *Xcc* suspension (1 × 10^5^ cfu mL^-1^) was subsequently inoculated. CBC development was assessed at 10 dpi, and both the disease severity (DS) and lesion size (LS) of the diseased spots were used for the assessment of CBC resistance. The DS was calculated as previously described^51,70^. CBC resistance was further evaluated through *Xcc* infiltration assays (1 × 10^5^ cfu mL^-1^), and canker symptoms were imaged at 10 dpi.

### Biochemical analysis

The activities of CIII Prx, SOD, CAT and GST and the concentrations of H_2_O_2_, O_2_^.−^, MDA, and lignin were measured via SinoBestBio assays (Shanghai, China). The experiments were repeated three times, and the results are shown as the means ± SEs.

### RNA isolation, cDNA synthesis and qRT-PCR assay

Miniprep kits (AidLab) were used for RNA isolation, and cDNA was synthesized using TaKaRa kits. qRT-PCR was performed using QuantStudio 7. The values were normalized to the *CsActin* levels (GenBank accession: GU911361.1, CAP ID: Cs1g05000) obtained using F_Actin_ (CATCCCTCAGCACCTTCC) and R_Actin_ (CCAACCTTAGCACTTCTCC). The qRT-PCR parameters were as follows: 95 °C for 5 min followed by 40 cycles of 95 °C for 10 s and 56 °C for 30 s. The reaction mixtures (total volume of 12 μL) contained 50 ng of cDNA, 0.5 μM primers and 6 μL of the PCR mix. The relative gene expression levels were assessed using the 2^-∆∆CT^ method^71^. NCBI was used for qRT-PCR primer design (Supplementary Table S3). The data are presented as the means from three independent biological repeats.

### Statistics

The data were analyzed using SPSS V22. Gene expression was compared by analysis of variance (ANOVA). The statistical significance was analyzed by Fisher’s LSD test. *P < 0.05 and **P < 0.01 indicate significant and extremely significant differences, respectively. The plant lines were compared using Tukey’s HSD test (*P* = 0.05).

## Discussion

CIII Prxs belong to a plant-specific multigene family that promotes disease resistance^18,33,34^, lignification, the flexibility of cell walls and suberization^29,30^. In sweet orange, 72 CIII Prxs have been identified^28^. The expression of each isoform varies across tissues and can be influenced by environmental factors, which suggests that different peroxidase isoenzymes regulate distinct processes^72^. The distribution of enzymes to either the cell walls or vacuoles and their destinations reflect their specific functions^31^. In CBC-resistant and CBC-susceptible varieties, *CsPrx25* exhibits altered expression patterns (Fig. 2d–f), which suggests its role during CBC development. The importance of CIII Prxs for the resistance of plants to pathogenic diseases was identified through reverse genetics. CIII Prxs mediate innate resistance both passively and actively^6^. HvPrx40^40^ and TaPrx10 ^39,41^ enhance the resistance of wheat against wheat powdery mildew. Here, CsPrx25 was found to mediate protection against *Xcc* pathogenesis, which confirmed its role as a CIII Prx and further highlighted the importance of this family in pathogen immunity in sweet orange. We explored its functional role using overexpression strategies and found that CsPrx25 strongly conferred CBC resistance to the transgenic plants (Fig. 3g–j).

Oxidative bursts, particularly the production of H_2_O_2_ and O_2_^.−^, are common innate responses in plant cells in response to pathogen infection^38^. As key enzymes for ROS homeostasis in plants, CIII Prxs have multiple functions and are proposed to serve as key regulators of the extracellular H_2_O_2_ and O_2_^−^ levels depending on peroxidative cycles (ROS scavenging) or hydroxylic cycles (ROS production)^73^. Plant defense responses are governed by the ROS levels and peroxidase-generated radicals, which mediate cell wall reinforcement, damage repair^4,6^ and apoptotic responses to induce plant resistance^5,6^. In this study, the molecular mechanisms of CsPrx25 were explored. Based on our analysis of ROS homeostasis and enzymatic antioxidant activities in the transgenic plants, we concluded that *CsPrx25* overexpression enhances CIII Prx activities and leads to a simultaneous improvement in the H_2_O_2_ and O_2_^.−^ content (Fig. 5a, b). In plants, the HR is directly related to plant disease resistance and represents the classic response to pathogen infection^6^. These reactions lead to both rapid and localized necrosis of the infected tissues and thus prevent the spread of infection^56,57^. H_2_O_2_ is key to the HR and is related to programmed cell death (PCD) in infected plants^58^. To investigate the relationship between the CBC resistance induced by CsPrx25 and the HR, we assessed the HR of the transgenic plants before and after *Xcc* infection (Fig. 5d). HSR203 is upregulated by plant HRs and is used as a marker for the HR levels^56,58^. In this study, the links among CsPrx25 activity, ROS content and HR level were established. Cell wall lignification was further shown to mediate CBC resistance, which was also demonstrated in rice due to the enhancement in *Xanthomonas oryzae* resistance conferred by CIII Prx-mediated lignification^73^.

Due to the evolutionary diversity and functional diversity of CIII Prxs, different studies have drawn different links between CIII Prx and disease resistance. Increased LePrx06 makes tomato more susceptible to *Pseudomonas syringae* infection. In contrast to CsPrx25, the suppression of *LePrx06* can enhance resistance to this pathogen^74^. Long-term studies of the relationship between the ROS levels and the development of CBC have revealed increased peroxidase activity and thus a reduced ROS content. Furthermore, the reduction in the ROS levels was associated with CBC resistance. These effects parallel the overexpression of *MdATG18a* and can enhance resistance to *Diplocarpon mali* infection via H_2_O_2_ scavenging^75^. Cybrids of grapefruit with a kumquat plastid genome exhibit increased CBC resistance through an early upregulation of ROS-controlling genes upon *Xcc* infection^76^. These findings illustrate potential links between ROS homeostasis mediated by plastid ROS-controlling genes and *Xcc* resistance. However, this study revealed that CsPrx25 is an apoplast-localized enzyme rather than a plastid enzyme (Fig. 2b, c), and this knowledge expands the list of ROS-controlling enzymes that can upregulate CBC resistance.

In this study of CsPrx25, regulation of the ROS levels by CsPrx25 and improvements in HR sensitivity were the major mechanisms through which transgenic citrus developed resistance to CBC. Although *CsPrx25* overexpression greatly improved the resistance of Wanjincheng to CBC, *CsPrx25*-overexpressing Wanjincheng cells were still not as resistant as Calamondin cells, which might be due to the fact that Calamondin also has other mechanisms to maintain an even higher level of CBC resistance. Anyway, this study explores new insights into the mechanisms of CIII Prxs in CBC resistance and provides potential clues for breeding CBC-resistant citrus.

## Supplementary information

Supplementary Figures

Supplementary Tables.xlsx
