# Supplementary material for: CsPrx25, a class III peroxidase in Citrus sinensis, confers resistance to citrus bacterial canker through the maintenance of ROS homeostasis and cell wall lignification
Source: Hortic Res. 2020 Dec 1;7:192. doi: 10.1038/s41438-020-00415-9 (PMC7705758; doi:10.1038/s41438-020-00415-9)

**Fig. S1. CsPrx25 expression in response to drought.** The expression in Wanjincheng (filled bars) and Calamondin (open bars) was assayed. The fresh leaves were placed in a dry, ventilated place at 28 °C for 48 h, and samples were collected at 0, 6, 12, 24, 36, and 48 h. The relative expression levels were measured by qRT-PCR and normalized to the CsActin levels. The data were analyzed using Tukey’s HSD test (*P*=0.05; n=3).


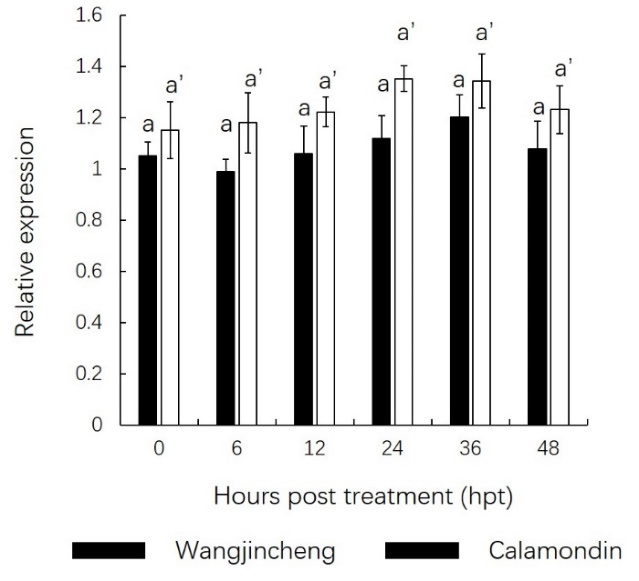

Supplement: Supplementary file 1 — Supplementary Figures [file 41438_2020_415_MOESM1_ESM.docx]
